# Assessment of the Concentration of Bone Metabolism Markers: Sclerostin and FGF-23 in Children with Idiopathic Nephrotic Syndrome Treated with Glucocorticosteroids

**DOI:** 10.1155/2019/9698367

**Published:** 2019-07-01

**Authors:** Agnieszka Pukajło-Marczyk, Anna Jakubowska, Agnieszka Bargenda-Lange, Katarzyna Kiliś-Pstrusińska, Danuta Zwolińska

**Affiliations:** Department of Pediatric Nephrology, Wroclaw Medical University, Borowska 213, Wroclaw 50-556, Poland

## Abstract

Recurring nature of idiopathic nephrotic syndrome (INS) and steroid dependence imply a long-term treatment with glucocorticosteroids (GCSs), which increases the risk of bone metabolism disorders. The search for new markers of that process is essential. The aims of this study were to assess the concentrations of sclerostin (Scl) and fibroblast growth factor-23 (FGF-23) in the plasma of children with INS and compare Scl and FGF-23 to existing markers of bone metabolism, mainly parathyroid hormone (PTH). The study involved 70 children, 50 with INS and 20 healthy children. Patients with INS were divided into 4 groups depending on the number of relapses and applied therapy. Significantly higher concentrations of FGF-23 and Scl were found in all patient groups with INS compared to the control group, and increase in the concentrations of examined parameters depending on the number of NS relapses was showed. In patients from the group with numerous relapses, higher concentrations of FGF-23 and Scl in the relapse phase than those in the remission phase were found. We observed positive correlation in these proteins with parathyroid hormone. Positive correlation of FGF-23 and Scl in the examined group was noted. Children having relapsing INS treated with steroids have higher levels of Scl and FGF-23 that can indicate the bone metabolism disorders. The significance of these observations requires further research.

## 1. Introduction

In the treatment of idiopathic nephrotic syndrome (INS) in children, glucocorticosteroids (GCSs) remain the medication of choice. Recurrent nature of this disease and steroid dependence imply a long-term therapy and probably increased risk of bone metabolism disorders. To this time, pediatrics population has not been analyzed in the context of changes in bone parameters during steroid therapy.

The mechanism of GCS impact on bone tissue is complex and depends on the duration of therapy. In the early period of treatment, bone resorption may be observed as a result of increased number and prolonged survival time of osteoclasts [1].

In the case of long-term steroid treatment, a reduction in the number and impairment of the function of osteoblasts occur [2], which results in the inhibition of bone formation process. GCS suppresses the differentiation of the bone marrow stromal cells in osteoblasts, through inhibiting the signaling pathway of Wnt/B-catenin [3] and through promoting the transformation of stromal cells into adipocytes [4].

The changes in bone metabolism due to glucocorticosteroid treatment also result from inhibition of transcription of the insulin-like growth hormone (IGF-1) [5], reduction of intestinal calcium absorption, renal calcium reabsorption, modulation of the secretion of parathyroid hormone (PTH), and reduction of the secretion of growth hormone and gonadotropins [6].

Recent studies suggest that the analysis of basic biochemical parameters does not allow an early assessment of bone metabolism disorders. Therefore, the search for new biochemical markers is underway, with sclerostin (Scl) and fibroblast growth factor-23 (FGF-23) as promising candidates.

Scl is a protein encoded by SOST gene, located on chromosome 17q12-q21, synthesized mainly by osteocytes. It affects bone formation by inhibiting the Wnt signaling pathway in osteoblasts. In studies on mice with induced Scl deficiency, Ryan et al. observed the increase in bone mass due to increased activity of osteoblasts with simultaneous decrease in activity of osteoclasts [7].

FGF-23 is a circulating secretory protein of molecular weight of 32 kDa, encoded by the FGF-23 gene, mainly expressed in osteocytes [8, 9]. The results of the conducted studies concerning the impact of FGF-23 factor on bone tissue are inconclusive; however, it has been shown that FGF-23 regulates osteoblast differentiation and its absence impairs skeletal mineralization, despite normal or increased levels of calcium and phosphorus [10, 11]. PTH and FGF-23, strongly inhibit proximal tubule phosphate reabsorption by stimulating NaPi-IIa endocytosis and its lysosomal degradation [12–14]. The biologic activity of FGF-23 on the proximal tubule requires the presence of klotho, expressed mainly in the distal tubule. Klotho acts as an obligatory cofactor for binding between FGF-23 and the FGFR receptor downstream signaling [15, 16].

Regulation of the FGF-23 concentration in the serum is affected by a number of factors, among others: active form of vitamin D_3_, calcium, and phosphates [17, 18]. Scl stimulates the secretion of FGF-23 [19]. Literature data suggest that increase of plasma concentrations of FGF-23 contributes to diminished bone mineral density [20] and may be a predictor of the increased risk of fractures [21].

The aims of this study were to assess the concentrations of Scl and FGF-23 in the plasma of children with INS treated with GCS and compare Scl and FGF-23 to existing markers of bone metabolism, mainly PTH.

## 2. Material and Methods

Seventy patients were enrolled in the study, 50 children with INS and 20 controls. The patients were recruited from January 2010 to December 2015. INS diagnosis was made based on the criteria of the International Study of Kidney Disease in Children (ISKDC) [22]. Relapse was recognized when urine protein-to-creatinine ratio (uPCR) was ≥2000 mg/g (≥200 mg/mmol).

Remission was defined when uPCR < 200 mg/g (<20 mg/mmol). The patients with INS (15 girls, 35 boys) were divided into 4 groups depending on the applied therapy and the numbers of relapses.

Group I consisted of 8 children with first occurrence of INS treated in accordance with the KDIGO recommendations. Group II consisted of 7 children during relapse, with number of relapses from 4 to 8, treated only with GCS (2 mg/kg/24 h). Group III included 15 patients with more than 8 relapses, treated with corticosteroid-sparing agents and high doses of GCS (2 mg/kg/24 h). Group IV consisted of 20 children in remission treated with corticosteroid-sparing agents and low doses of GCS (up to 5 mg/48 h), with chronic GCS therapy in the past.

The control group consisted of 20 children (11 girls, 9 boys) with primary monosymptomatic nocturnal enuresis or with negative observation for urinary tract abnormalities.

All patients had normal renal function throughout the study. None of them showed clinical evidence of infection or had elevated concentrations of markers of inflammation.

Informed consent was obtained from the parents and subjects if they were over 16 years of age.

The study protocol in accordance with the Declaration of Helsinki was approved by the Bioethics Committee at the Wroclaw Medical University (opinion KB-199/2009).

Blood samples were drawn from the cubital vein after an overnight fast, during routinely performed laboratory tests. Samples were clotted for 30 min, centrifuged at room temperature for 10 min, and then plasma was stored at −80°C until assayed. Plasma Scl and FGF-23, serum PTH, calcium, and phosphorus were assayed.

In groups I and III, the concentrations of FGF-23, sclerostin, and PTH were measured twice in the same patient, during relapse and in remission. The time of the second evaluation was different for different patients and depended on the time required for achieving remission. In groups II and IV, the assays were performed only one time, in relapse and remission, respectively. Calcium and phosphorus were measured in relapse of INS (groups I–III) and in remission (group IV).

The plasma concentrations of Scl and FGF-23 were evaluated by ELISA with the use of sets manufactured by Wuhan Science EIAab Science Co., China (Scl: reagent kit E1713h; FGF-23: reagent kit E0746h). The assay used in the study measures the intact FGF-23. Measurements were performed in accordance with the instructions provided by the manufacturer. The evaluations were performed twice, and then the average of the obtained results was calculated. The sensitivity of the method was 0.31 ng/ml and 15.6 pg/ml for Scl and FGF-23, respectively.

The serum creatinine, calcium, phosphorus, and PTH were assessed by standard laboratory techniques. Estimated glomerular filtration rate (eGFR) was calculated according to the Schwartz formula [23].

## 3. Statistical Analysis

The results are expressed as median values, quartiles (first and third), and range (minimum and maximum values). Due to the small number of patients, verification of the hypothesis of median value equality in regard to the studied parameters in individual groups was carried out using nonparametric Kruskal-Wallis rank sum test. Verification of the hypothesis of median value equality in regard to the studied parameters in individual dependent samples (e.g., relapse-remission) was conducted using nonparametric Wilcoxon pair sequence test. For discrete parameters (gender), the incidence of feature occurrence in the groups was analyzed by *χ*^2^ test with Yates' correction and with one degree of freedom or, when the expected value in a cell was less than 5, by Fisher's test. Relations between parameters were defined by Pearson's correlations coefficient *r*. A *p* value of <0.05 was considered to be statistically significant. The statistical analysis was performed using a computer statistical software package EPIINFO ver. 7.1.1.14 (dated 2-07-2013). The results are presented in tables and graphs.

## 4. Results

Basic demographic data of patients are shown in Table 1.

Group I is comprised of children with first occurrence of INS treated with GCS (2 mg/kg/24 h); group II is INS children during relapse, with the number of relapses from 4 to 8, treated only with GCS (2 mg/kg/24 h); group III is INS patients with more than 8 relapses, treated with corticosteroid-sparing agents and GCS (2 mg/kg/24 h); and group IV is children in remission treated with corticosteroid-sparing agents and low doses of GCS (up to 5 mg/48 h), with chronic GCS therapy in the past.

The plasma concentrations of Scl and FGF-23 in children with INS were significantly higher than those in the control group (Table 2).

**Figure 1:**
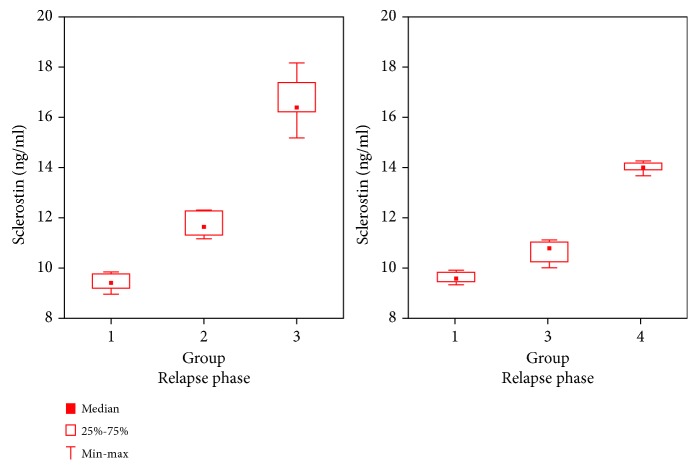
The plasma concentrations of Scl in the studied groups during relapse and remission.

**Figure 2:**
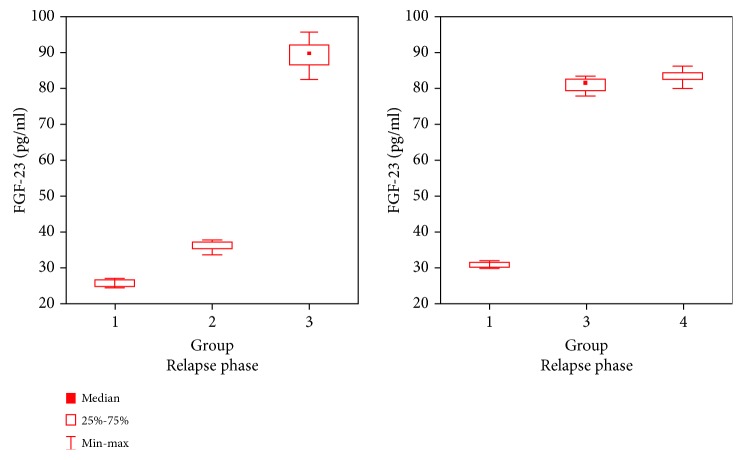
The plasma concentrations of FGF-23 in the studied groups during relapse and remission.

The values of plasma concentrations of Scl and FGF-23 differed between examined groups of children with INS and increased along with the number of relapses (Figures 1 and 2).

The plasma concentrations of Scl in patients from group III were higher during relapse than those in remission (*p* = 0.005), whereas no such difference was observed in group I (*p* = 0.67). In group I, the plasma concentrations of FGF-23 during relapse were lower than those in remission (*p* = 0.0117), whereas in group III, the concentrations of FGF-23 during relapse were higher compared to those observed in remission (*p* = 0.005). In group IV, plasma concentrations of Scl and FGF-23 were lower than those in group III during relapse (*p* < 0.0001), but they exceeded the values observed in that group in remission (*p* = 0.012).

The serum concentrations of PTH in children with INS, both during relapses and in remissions, were significantly higher than those in the control group (Table 3). The values of PTH concentrations differed between the examined groups of children with INS and increased along with the number of relapses (Table 3).

In groups I and III, concentrations of PTH during relapse were higher than those in remission (*p* = 0.0117 and *p* = 0.005, respectively). In group IV, concentration of PTH exceeded the values observed in group III in remission phase (*p* ≤ 0.001).

In groups I, II, and III, the calcium concentrations were evaluated only in relapse and were lower compared to the values observed in the control group (*p* < 0.0074). No difference was observed between group IV and the control group. There were no statistically significant differences between concentrations of phosphorus in all groups of patients with INS versus the control group.

The Scl and FGF-23 levels in plasma correlated significantly with the values of PTH in serum during relapse (*r* = 0.95, *p* < 0.0001 and *r* = 0.97, *p* < 0.0001, respectively) and in remission (*r* = 0.86, *p* < 0.0001 and *r* = 1.00, *p* < 0.0001, respectively). There was no correlation found between the studied markers and concentrations of calcium and phosphorus. The values of Scl and FGF-23 correlated positively with each other in groups of children with INS, both during relapse (*r* = 0.90, *p* < 0.0001) and in remission (*r* = 0.87, *p* < 0.0001).

## 5. Discussion

In the present study, we investigated for the first time the plasma concentrations of Scl and FGF-23 in children with INS chronically treated with GCS.

Human skeleton during the growth period may be particularly vulnerable to adverse effects of long-term steroid therapy. The studies on children with INS showed that there is a reverse correlation between bone density and cumulative dose of GCS treatment [1]. As a result, there is a need to assess new markers of mineral and bone metabolism in INS children treated with GCS.

Our investigation revealed that the plasma concentration of Scl in patients with INS increases along with the duration of GCS therapy, the exponent of which was the number of disease relapses. That observation is consistent with results of study by Yao et al. In mice, the duration of the steroid treatment positively correlated with expression of Scl-encoding gene SOST, associated with the inhibition of activation and maturation of osteoblasts [24]. Data concerning connection between Scl and GCS therapy are very scarce.

On the other hand, the influence of Scl on bone metabolism has been the subject of various studies. In a rat model of postmenopausal osteoporosis due to ovariectomy, treatment with a Scl antibody (Scl-Ab) increased bone mass at all skeletal sites and completely prevented bone loss associated with estrogen deficiency [25]. The study of Ominsky et al. [26] conducted on monkeys with intact gonads also revealed clear anabolic effect of Scl-Ab, with marked dose-dependent increases in bone formation on trabecular, periosteal, endocortical, and intracortical surfaces, in a short period of time after the administration of Scl-Ab. Those results stand in contradiction with the study of Polyzos et al. [27], in which the serum concentrations of Scl were lower in the group of postmenopausal women with osteoporosis than those in the group with normal bone density. Dutch authors, in turn, demonstrated that diseases characterized by high bone turnover, i.e., Paget's disease, or in metastatic prostate cancer, are associated with significantly increased Scl concentrations in the serum [28]. Cejka et al. noted that in patients on hemodialysis, the serum concentrations of Scl were significantly higher compared to those observed in sex- and age-matched healthy controls. Additionally, the concentrations of Scl correlated positively with vitamin 25(OH)D_3_ and calcium and negatively with intact PTH concentrations [29]. Based on the above results, the role of Scl in mineral and bone metabolism remains unclear.

Another marker examined by us was FGF-23. We have found that FGF-23 concentrations in the plasma of patients with INS, similar as Scl concentrations, increased along with the increase in exposure to GCS.

So far, FGF-23 in the pediatric population has been assessed mainly in children with chronic kidney disease (CKD). FGF-23 has been established as a reliable marker of bone metabolism disorders due to CKD [11].

Portale et al. studied plasma concentrations of FGF-23 in children with CKD resulting from different causes [30]. They stated a significantly higher concentration of FGF-23 in patients, in whom the underlying cause of CKD was glomerulopathy. Similarly, Lundberg et al. found that circulating FGF-23 is associated with albuminuria and CKD progression in patients with IgAN [31]. Bacchetta et al. studied the effects of various parameters on the plasma concentration of FGF-23. They showed that the concentration of this protein is not affected by gender, but its values decrease with age. Moreover, they found that treatment with the use of GCS is correlated with the increase of FGF-23 concentration [32]. It is in agreement with our observations.

de Seigneux et al. demonstrated that proteinuria impairs tubular phosphate excretion, due to inhibiting effect of FGF-23 on NaPi-IIa expression in tubules. The studied group consisted of children with nephrotic syndrome with normal GFR and patients with deteriorated kidney function. In the group of children with nephrotic syndrome, concentrations of FGF-23 were significantly higher in the relapse than in the remission, which is also consistent with our results. The study conducted by the same authors on proteinuric animals suggests that differences between concentrations of FGF-23 during relapse and remission result from decreased cellular response to FGF-23. Despite high plasma levels of FGF-23, phosphorylation of FRS2a, a major downstream target of FGF receptor, was decreased. This altered FGF-23 signaling appears to be mainly related to decreased klotho expression by proteinuria, independent of GFR [33].

Based on results of our study, we assume that proteinuria might not be the only factor affecting the concentrations of FGF-23. Along with a growing number of relapses, the concentrations of FGF-23 increased. In group of patients remaining in remission, treated with cyclosporine A, with previous history of repetitive GCS therapy, the concentrations of FGF-23 continued to stay high. We suggest that long-term GCS therapy may have a crucial impact on observed aberrations of FGF-23.

In our investigation, we showed positive correlation between plasma concentrations of Scl and FGF-23, as well as positive correlation of these parameters with PTH, which is known marker of mineral metabolism. Interestingly, in the group of patients with the first occurrence of nephrotic syndrome, the concentrations of FGF-23 were higher during remission than those during the active phase of the disease. On contrary, the concentrations of PTH were lower in remission. We did not observe any significant differences in the concentrations of sclerostin within that group. In the group of patients with multiple relapses, the concentrations of sclerostin, FGF-23, and PTH were significantly higher during relapse than those in remission. We assume that observed changes reflect the aggravation of bone metabolism disorders due to re-initialization of high dose GCS regimen.

These observations can indicate that Scl and FGF-23 are important “players” in bone metabolism disturbances due to GCS therapy in INS.

## 6. Limitation of the Study

Our study has few limitations. The small size of the study groups and a cross-sectional character of the study allow to detect associations; however, prospective studies with larger sample size are necessary to validate our data and to determine the cause-effect relationship between GCS therapy, FGF-23, and Scl. Supplementation of vitamin D_3_, which we regimen according to recommendations during high dose of GCS therapy, could influence results in groups I and III.

Additionally, it would be advisable to compare the usefulness of the studied parameters with other methods of skeleton assessment, e.g., densitometry and ultrasonography, in patients chronically treated with GCS.

## 7. Conclusions

All we can conclude is that children having relapsing INS treated with steroids have higher levels of Scl and FGF-23 that can indicate the bone metabolism disorder. The significance of these observations requires further research on a larger group of patients.

## Figures and Tables

**Table 1:** The patient characteristics.

	Control group (*n* = 20)	Patients with INS			
		Group I (*n* = 8)	Group II (*n* = 7)	Group III (*n* = 15)	Group IV (*n* = 20)
Age (years)	8.0 (7.00, 13.00)	3.75 (3.25, 7.5)	8.0 (5.5, 11)	12.5 (10, 16)	11.5 (7.6, 13.8)

Gender	11 girls	3 girls	2 girls	7 girls	3 girls
	9 boys	5 boys	5 boys	8 boys	17 boys

Data are presented as median values and interquartile ranges.

**Table 2:** The plasma concentrations of Scl and FGF-23 in the studied groups.

		Scl (ng/ml) M (25Q, 75Q) min-max	FGF-23 (pg/ml) M (25Q, 75Q) min-max
Control group (*n* = 20)		4.92 (4.75, 5.04) 4.46-5.21	6.93 (6.54, 7.17) 6.24-7.72

Group I (*n* = 8)	First occurrence	9.41 (9.18, 9.75)^a^ 8.90-9.78	25.9 (24.8, 26.5)^a^ 24.2-27.1
	Remission	9.60 (9.43, 9.75) 9.25-9.86	30.8 (30.2, 31.2) 29.6-31.7

Group II (*n* = 7)	Relapse	11.7 (11.3, 12.2)^a^ 11.1-12.2	35.4 (35.0, 37.1)^a^ 33.6-37.7
	Remission	—	—

Group III (*n* = 15)	Relapse	16.4 (16.2, 17.4)^a^ 15.1-18.2	89.7 (86.2, 91.9)^a^ 82.4-95.4
	Remission	10.79 (10.21,11.0) 9.92-11.07	81.8 (79.8, 82.7) 77.9-83.6

Group IV (*n* = 20)	Relapse	—	—
	Remission	14.0 (13.9, 14.2)^a^ 133.7-14.3	82.9 (82.2, 84.6)^a^ 80.2-86.2

Data are presented as median values, quartiles (first and third), and range (minimum and maximum values). ^a^*p* < 0.0001 INS group versus the control group.

**Table 3:** The concentrations of PTH, calcium, and phosphorus in the studied groups during relapse and remission.

		PTH (pg/ml) M (25Q, 75Q) min-max	Calcium (mg/dl) M (25Q, 75Q) min-max	Phosphorus (mg/dl) M (25Q, 75Q) min-max
Control group (*n* = 20)		82.2 (80.8,83.3) 80.2-84.4	9.91 (9.80, 9.99) 9.54-10.42	4.73 (4.60, 5.10) 3.54-5.40

Group I (*n* = 8)	First occurrence	108.0 (105.7,109.7) 104.5-111.0	8.61 (8.10, 8.82) 8.00-9.38	4.49(2.80, 5.50) 1.90-6.34
	Remission	98.6 (98.0, 99.3) 96.8-100.6	—	—

Group II (*n* = 7)	Relapse	117.2 (116.1, 118.9) 114.4-119.6	9.18 (8.40, 9.64) 7.84-10.03	4.67 (4.00, 5.00) 2.16-5.30
	Remission	—	—	—

Group III (*n* = 15)	Relapse	138.0 (137.4, 138.9) 136.7-139.0	8.40 (8.00, 9.60) 7.44-9.90	5.00 (3.90, 5.50) 3.30-7.30
	Remission	123.2 (123.0, 124.3) 122.4-124.4	—	—

Group IV (*n* = 20)	Relapse	—	—	—
	Remission	132.2 (131.5, 133.0) 130.3-133.6	9.78 (9.24,10.00) 8.01-10.50	4.30 (4.10, 4.68) 3.90-5.71

Data are presented as median values, quartiles (first and third), and range (minimum and maximum values).

## Data Availability

The database used to support the findings of this study is available from the corresponding author upon request.
